# New Catechol Derivatives of Safrole and Their Antiproliferative Activity towards Breast Cancer Cells

**DOI:** 10.3390/molecules16064632

**Published:** 2011-06-03

**Authors:** Alejandro Madrid Villegas, Luis Espinoza Catalán, Iván Montenegro Venegas, Joan Villena García, Héctor Carrasco Altamirano

**Affiliations:** 1 Departamento de Química, Universidad Técnica Federico Santa María, Av. España N° 1680, Valparaíso, Chile; Email: luis.espinozac@usm.cl (L.E.C.); 2 Centro de Investigaciones Biomedicas (CIB), Centro Regional de Estudios en Alimentos Saludables, Escuela de Medicina, Universidad de Valparaíso, Creas, Av. Hontaneda N° 2664, Valparaíso, Chile; 3 Departamento de Ciencias Químicas, Universidad Andrés Bello, Campus Viña del Mar, Los Fresnos N° 52, Viña del Mar, Chile; Email: hcarrasco@unab.cl (H.C.A.)

**Keywords:** antiproliferative activity, catechol, synthesis

## Abstract

Catechols were synthesized from safrole. Nine derivatives were prepared and assessed for antiproliferative effects using different human cell lines. The *in vitro* growth inhibition assay was based on the sulphorhodamine dye to quantify cell viability. The derivatives 4-allylbenzene-1,2-diol (**3**), 4 4-[3-(acetyloxy)propyl]-1,2-phenylene diacetate (**6**) and 4-[3-(acetyloxy)propyl]-5-nitro-1,2-phenylene diacetate (**10**) showed higher cytotoxicity than the parent compound **2** in tests performed on two breast cancer cell lines (MCF-7 and MDA-MB-231). The IC_50_ values of 40.2 ± 6.9 μM, 5.9 ± 0.8 μM and 33.8 ± 4.9 μM, respectively, were obtained without toxicity towards dermal human fibroblast (DHF cells).

## 1. Introduction

Safrole is a colorless or slightly yellow oily liquid that has been commonly used in cosmetics and as a food flavoring, extracted as sassafras oil from the root-bark or the fruit of sassafras (Lauraceae) plants, or synthesized from other compounds linked to methylenedioxy [1,2]. *In vitro*, a depletion of glutathione (GSH) was observed by the addition of safrole to hepatocytes [3,4]. In 1994, Bolton pointed out that hydroxychavicol could be further biotransformed to *o*-quinone through a 2-electron oxidation *in vitro* [4]. This characterization of the GSH conjugates revealed that the *o*-quinone moiety undergoes regioselective attack by GSH at C6 on the cyclohexadienedione ring. Under neutral or acidic conditions, sulfur nucleophiles add primarily 1,6 to 4-substituted *o*-benzoquinones. As catechols are readily oxidized, the conversion of l-allyl-3,4-dihydroxybenze (HC) to an *o*-quinone or *p*-quinone methide (QM) may contribute to the cytotoxic and/or genotoxic activity of safrole. In previous studies, the effects of safrole oxide derivatives on cellular growth and apoptosis in vascular endothelial cells (VEC) were evaluated [5]. In this study, the authors concluded that safrole oxide induced apoptosis by up-regulating the expressions of transmembrane protein (Fas) and by reducing the activity of reactive oxygen species (ROS). The authors also reported that safrole oxide can inhibit angiogenesis. This is induced by cancer cells *in vitro* and *in vivo* [6,7]. It has been recently reported that safrole derivatives (catechols) are more toxic than safrole against MCF-7 and MDA-MB-231 cells [8]. The safrole derivatives proved to be more toxic than safrole itself against the selected cancer cells and not to cause damage in dermal human fibroblast cells. The formation of catechols from safrole with AlCl_3_ was favoured by the presence of the electron attractor group (NO_2_) in the molecule. These results suggest that derivatives of safrole bearing a nitro group and a hydroxyl group can present an antiproliferative/cytotoxic effects on cells. The screening *in vitro* provides preliminary data to select compounds with potential antineoplastic properties. Safrole (**1**) and its synthetic derivatives **3-11** were tested *in vitro* for antiproliferative effects on two human tumor breast cancer cell lines (MDA-MB-231, MCF-7), and on one dermal human fibroblast cell line (DHF). The sulforhodamine B assay was used to quantify cell viability.

## 2. Results and Discussion

### 2.1. Chemistry

**Scheme 1:**
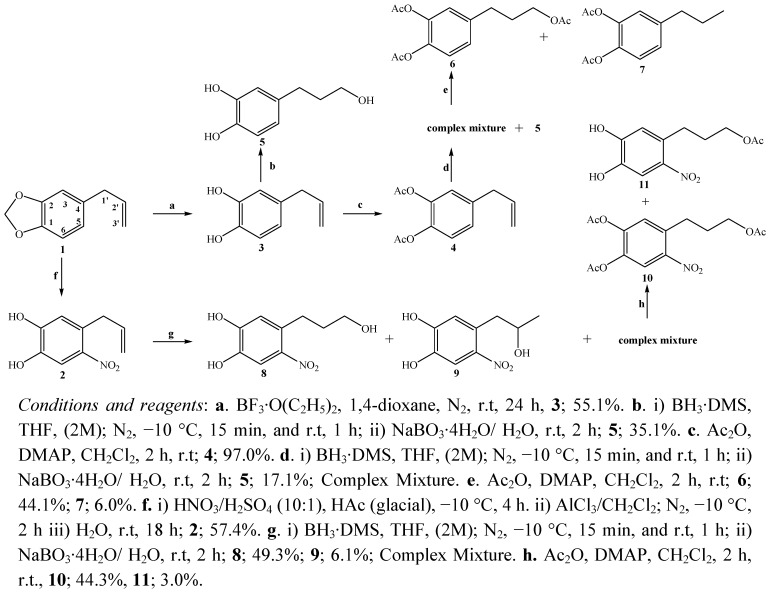
General scheme of synthesis of derivatives of safrole.

The catechol **3** was obtained and synthesized by the BF_3_•O(C_2_H_5_)_2_ solution method [9] in 55.1% yield, higher that the reported previously for compounds **2** [8,10,11,12,13] (Scheme 1). Oxidative hydroboration of the side chain in both catechols led to a hydroxyl group in C-3’. The side chains of the catechols were oxidized using BH_3_·DMS/THF/NaBO_3_·4H_2_O/H_2_O as previously described for other alkenes and alkynes [14,15,16,17,18,19]. The primary alcohols **5** and **8** were obtained in 35.1% and 49.3% w/w yields and the secondary alcohol **9** with 6.1% yield (Scheme 1). Hydroboration of acetylated catechol **4** produced the triol **5** (a product obtained due to the alkaline pH generated in the oxidation process) [8] plus a polar complex mixture, which after acetylation gave acetylated triol **6** plus the catechol with a saturated side chain **7** (Scheme 1). All the compounds were characterized by NMR, IR and MS spectral data.

Cleavage of the methylenedioxy ring of safrole took place during the conversion into the catechol **3 (**55.1% yield) using the new BF_3_•O(C_2_H_5_)_2_ solution method [12,13]. In the ^1^H-NMR spectrum, the absence of the methylenedioxy singlet between 5.90–6.05 ppm confirmed the presence of catechol, where now two broad signals at δ = 5.29 (b.s., 1H) and δ = 5.21 (b.s., 1H) are observed. The MS spectrum of the diacetate derivative **3 ** showed two peaks at *m/z* 175 (26.6%) and 116 (24.3%) presumably due to the loss of two acetate groups. The first oxidative hydroboration of the side chain in catechol **2** and the subsequent hydroboration of acetylated catechol **3** was carried out to obtain the C-3’ alcohol **5** with different yields in both cases (35.1% and 17.1%, respectively). Additionally in the hydroboration of **4** a complex mixture was obtained that was derivatized by acetylation, to give triacetate compound **6** and the acetylated catechol **7** with a saturated side chain**.**

In the IR spectrum of compound **5 ** the broad absorption at 3398 cm^−1 ^was assigned to a hydroxyl group, whereas in the ^1^H-NMR spectrum three singlet signals at δ = 7.75 ppm (s, 1H), 7.73 ppm (s, 1H) and 5.69 ppm (b.s, 1H) for three hydroxyl groups were observed. Finally, using the experimental data obtained from the NMR spectra, the structure of triacetyl derivative **6** was confirmed. 

On the other hand in the ^1^H-NMR spectrum of compound **7** three signals at δ = 2.57 ppm (t, 2H, *J* = 7.7 Hz), δ = 1.63 ppm (m, 2H) and δ = 0.95 ppm (t, 3H, *J* = 7.7 Hz) were observed, assigned to the hydrogens H-1', H-2' and H-3', respectively, corresponding these three signals to a typical *n*-propyl group. The ^13^C-NMR spectrum (in combination with ^13^C-DEPT-135) showed the presence of two CH_3_ signals at δ = 20.6 and 13.8 ppm; the first was assigned to the methyl groups of both acetates and second assigned to the terminal CH_3_ in the *n*-propyl side chain.

In the hydroboration of compound **2** both isomers, the primary and secondary alcohols, were obtained in 49% and 6.1% yield, respectively. In the ^1^H-NMR spectrum of compound **8**, three signals at δ = 4.39 (t, 2H, *J* = 6.0 Hz); 2.88 (m, 2H) and 1.79 (m, 2H) were assigned to H-3’, H-1’ and H-2’ in the saturated side chain, respectively. In the ^1^H NMR spectrum of isomer **9**, the presence of signal at δ = 4.12 ppm (m, 1H’) was assigned to a carbinolic hydrogen in the C-2’ position (secondary alcohol). Also the signal at δ = 1.33 (d, 3H, *J* = 6.6 Hz) was to assigned a terminal methyl group. Compound **11** was finally identified as follows: in IR spectrum the observed bands at 3351, 1739 and 1546 cm^−1^, were assigned to the OH, carbonyl and NO_2_ groups. The ^1^H-NMR spectrum showed a signal at δ = 2.94 ppm (s, 2H) assigned to two OH groups. The structure was confirmed by the ^13^C-NMR spectrum with two signals δ = 171.0 and 20.7 ppm, corresponding to a single carbonyl carbon and a methyl acetate group, respectively.

### 2.2. Biological Results

The *in vitro* cytotoxicity evaluation of compounds **3-11** synthesized from safrole and nitrated catechol **2** (see Scheme 1) indicated that the cell viability expressed as % *vs.* control vehicle (ethanol 0.1%) for the compounds synthesized was dose-dependant (µM). IC_50_ values of compounds **3-11**, safrole, catechol **2** and the reference compound daunorubicin are summarized in Table 1 as the micromolar concentration that produces 50% cell growth inhibition after 72 hours of drug exposure. 

**Table 1:** Cytotoxic activity of synthesized derivatives safrole compounds against two breast cancer cell lines, **MCF-7**, **MDA-MB-231** and a dermal human fibroblast cells, DHF, expressed as inhibitory concentration IC_50_.

Compound	IC _50_ (μM)		
	MCF-7	MDA-MB-231	DHF
**2**	**55.0 ± ** **7.11**	**37.5 ± ** **2.65**	**>100**
**3**	**98.4 ± 11.6**	**40.2 ± 6.9**	**>100**
**4**	**>100**	**54.7 ± 6.6**	**>100**
**5**	**>100**	**54.3 ± 4.1**	**>100**
**6**	**97.8 ± 12.4**	**5.9 ± 0.8**	**>100**
**7**	**>100**	**41.7 ± 7.4**	**>100**
**8**	**78.2 ± 8.9**	**57.3 ± 4.1**	**>100**
**9**	**>100**	**>100**	**>100**
**10**	**51.3 ± 2.1**	**33.8 ± 4.9**	**>100**
**11**	**63.8 ± 5.5**	**27.1 ± 4.4**	**>100**
**safrole**	**>100**	**>100**	**>100**
**Daunorubicin**	**0.19 ± 0.01**	**0.38 ± 0.06**	**14.3 ** **± ** **1.85**

The results indicate that derivative **10** is more toxic than safrole and **2** against MCF-7 cells. Compound **10** also shows higher toxicity against all the cancer cells tested than the parent compound and derivative **2**. The activity of compounds **3**, **4**, **5**, **6**, **7**, **8**, **9** and **11** was not significantly different from that of safrole. The compounds **6** and **10** have higher activity in MDA-MB-231 cells than in MCF-7 cells. On the other hand the catechol compounds **2** and **8** have a high activity than compounds **6** and **10**. The rest of the compounds have no significant differences with activity of safrole. However, daunorubicin is at least one hundred times more active as an anticancer compound against MDA-MB-231 cells and 100–200 times more effective towards MCF-7 cells than the safrole derivatives **3 ** and **4.** Nevertheless, it is important to emphasize that compound **6 ** has ten times less cytotoxic than daunorubicin against normal cells, an interesting feature. Safrole has been shown to become cytotoxic to various human cell types at higher concentrations (5–10 mM) [20,21]. Our work suggests that the derivatization of this compound generates potent anti-cancer compounds, as demonstrated by previously synthesized products as **2** and the new modifications as in **6** and **10**.

## 3. Experimental

### 3.1. General

Safrole was obtained commercially from Sigma-Aldrich and the chemical reagents were purchased (Merck or Aldrich) with the highest purity commercially available and were used without previous purification. IR spectra were obtained by using KBr pellets or thin films in a Nicolet Impact 420 spectrometer. Frequencies are reported in cm^−1^. Low resolution mass spectra were recorded on a Shimadzu QP-2000 spectrometer at 70 eV ionising voltage and are given as m/z (% rel. int.) ^1^H, ^13^C (DEPT 135 and DEPT 90). Some spectra were recorded in CDCl_3_ solutions and were referenced to the residual peaks of CHCl_3_, δ = 7.26 ppm and δ = 77.0 ppm for ^1^H and ^13^C, respectively; CD_3_COCD_3_ solutions were referenced to the residual peaks of CH_3_COCH_3_, δ = 2.04 ppm and δ = 29.8 ppm for ^1^H and ^13^C, respectively, on a Bruker Avance 400 Digital NMR spectrometer operating at 400.1 MHz for ^1^H and 100.6 MHz for ^13^C. Chemical shifts are reported in δ ppm and coupling constants (*J*) are given in Hz. Silica gel (Merck 200-300 mesh) was used for C.C. and silica gel plates HF-254 for TLC. TLC spots were detected by heating after spraying with 25% H_2_SO_4_ in H_2_O.

*Synthesis of 4-allylbenzene-1,2-diol* (**3**) *from* **1**: A BF_3_•O(C_2_H_5_)_2_ solution (0.3 mL, 2.39 mmol) was slowly added to a solution of safrole (**1**, 100 mg, 0.62 mmol) in anh.1,4-dioxane (10 mL) under an atmosphere of N_2_ with gentle stirring. The reaction was continued for 24 h. to r.t. After this the mixture was taken up in water and then extracted with ethyl acetate (3 × 50 mL). Later the layers were separated. The watery layer was discarded and the organic layer was washed to neutrality with a saturated solution of NaHCO_3_. The organic layer was dried over MgSO_4_, filtered, evaporated. Then it was absorbed on silica, chromatographed by CC eluting with mixtures of petroleum ether/EtOAc of increasing polarity (17.0:3.0→16.0:4.0) to give an orange oil identified as catechol **3** (51.3 mg, 55.1%); IR (cm^−1^): 3321 (O-H); 2917 (C-H); 1618 (C=C); 1430 (-CH_2_); 1045 (-C-O); 809 (-C-H). ^1^H- NMR: 6.78 (d, 1H, *J* = 8.0 Hz, H-6); 6.71 (d, 1H, *J* = 1.9 Hz, H-3); 6.62 (dd, 1H, *J* = 8.0, 1.9 Hz, H-5); 5.93 (ddt, 1H, *J* = 16.9, 10.2 and 6.8 Hz, H-2’); 5.29 (b.s., 1H, OH); 5.21 (b.s., 1H, OH); 5.05 (m, 2H, H-3’); 3.27 (d, 2H, *J* = 6.7 Hz, H-1’). ^13^C-NMR: 143.5 (C-2); 141.7 (C-1); 137.6 (C-2’); 133.2 (C-4); 121.0 (C-5); 115.7 (C-6); 115.6 (C-3); 115.3 (C-3’); 39.5 (C-1’).

*Synthesis of 4-allyl-1,2-phenylene diacetate* (**4**) *from* **3**: To a solution of **3** (0.30 g, 2.00 mmol) in dry CH_2_Cl_2_ (60 mL), DMAP (1.00 mg) and Ac_2_O (0.38 µL, 4.00 mmol) were added and the mixture was stirred at room temperature for 2 h. A cooled solution of 10% KHSO_4_ (approx. 50 mL) was then added to this mixture. The watery layer was discarded and the organic layer was washed to neutrality with a saturated solution of NaHCO_3_ and water. Then it was dried over MgSO_4_, filtered, evaporated and re-dissolved in CH_2_Cl_2 _(5 mL). Subsequently, it was absorbed on silica and chromatographed by CC with petroleum ether/EtOAc mixtures of increasing polarity (19.8:0.2→16.4:3.6) to give **4**, an amber oil (0.45 mg, 97.0); 39.4 (C-1’); 20.6 (2×CH_3_CO).

*Synthesis of 4-(3-hydroxypropyl)benzene-1,2-diol* (**5**) *from* **3**: The compound **3 **(150 mg, 1.0 mmol) cooled to −5 °C, under an atmosphere of N_2_, was slowly hydroborated with a 2.0 M solution of BH_3_·DMS/THF (0.25 mL) added dropwise at −10 °C over 15 min. Then the mixture was stirred at room temperature for 1 h. The resultant organoborane was oxidized by sodium perborate (0.95 g, 6.2 mmol) and water (100 mL). The mixture was stirred at room temperature for 2 h. Then, it was extracted with portions of ethyl ether (4 × 50 mL) and the layers were separated. The organic layer was dried over MgSO_4_, filtered, evaporated and re-dissolved in 5 mL of CH_2_Cl_2_. It was absorbed on silica, chromatographed by CC, eluting with mixtures of petroleum ether/EtOAc of increasing polarity (18.8:1.2→17.6:2.4) to give a brown oil identified as triol **5** (58.8 mg (35.1%); IR (cm^−1^): 3398 (O-H); 2931 (C-H); 1643 (C=C); 1467 (-CH_2_); 1031 (C-O); 890 (-C-H). ^1^H-NMR: 7.75 (s, 1H, OH); 7.73 (s, 1H, OH); 5.69 (b.s, 1H, OH); 6.68 (m, 2H, H-3 and H-6); 6.51 (dd, 1H, *J* = 8.1 and *J* = 1.8 Hz, H-5); 3.56 (t, 2H, *J* = 6.5 Hz, H-3’); 2.52 (t, 2H, *J* = 7.7 Hz, H-1’); 1.75 (m, 2H, H-2’). ^13^C-NMR: 145.2 (C-2); 144.6 (C-1); 134.7 (C-4); 120.3 (C-5); 117.2 (C-6); 116.1 (C-3); 63.1 (C-3’); 32.1 (C-2’); 30.4 (C-1’). 

*Synthesis of 4-[3-(acetyloxy)propyl]-1,2-phenylene diacetate* (**6**) *and 4-allyl-1,2-phenylene diacetate* (**7**) *from* **4**: The compound **4 **(250 mg, 1.1 mmol) cooled to −5 °C, under an atmosphere of N_2_, was slowly hydroborated with a 2.0 M solution of BH_3_·DMS/THF (0.25 mL) at −10 °C for 15 min was added dropwise. Then the mixture was stirred at room temperature for 1 h. The resultant organoborane was oxidized by sodium perborate (0.95 g, 6.2 mmol) and water (100 mL). The mixture was stirred at room temperature for 2 h. Then, it was extracted with portions of ethyl ether (4 × 50 mL) and the layers were separated. The organic layer was dried over MgSO_4_, filtered, evaporated and re-dissolved in 5 mL of CH_2_Cl_2_. It was absorbed on silica, chromatographed by CC, eluting with mixtures of petroleum ether/EtOAc of increasing polarity (18.8:1.2→17.6:2.4). Then two fractions were obtained: fraction I (4.8 mg, 17.1%) of compound **5** and fraction II (147.4 mg), a mixture of polar products. This mixture was dissolved in dry CH_2_Cl_2_ (50 mL), DMAP (1.02 mg) and Ac_2_O (0.40 mL, 4.23 mmol) were added and the mixture was stirred at room temperature for 2 h. A cooled solution of 10% KHSO_4_ (approx. 50 mL) was then added to this mixture. The watery layer was discarded and the organic layer was washed to neutrality with a saturated solution of NaHCO_3_ and water. Then it was dried over MgSO_4_, filtered, evaporated and re-dissolved in 5 mL of CH_2_Cl_2_. Subsequently, it was absorbed on silica and chromatographed by CC with petroleum ether/EtOAc mixtures of increasing polarity (19.8:0.2→17.8:2.2). Two products were isolated identified as the triacetylated **6** (139 mg, 44.1%) and the diacetylated **7** (15 mg, 6.0%). Compound **6**; IR (cm^−1^): 2925 (C-H); 1731 (C=O); 1561 (C=C); 1429 (-CH_2_); 1214 (C-O); 1076 (C-O); 896 (C-H). M.S. (m/z, %): 295 (25.7); 282 (18.4); 281 (64.2); 267 (11.5); 222 (18.7); 221 (80.6); 207 (33.6); 147 (78.6); 73 (100). ^1^H-NMR: 7.08 (m, 2H, H-5 and H-6); 7.01 (s, 1H, H-3); 4.10 (t, 2H, *J* = 6.5 Hz, H-3’); 2.68 (t, 2H, *J* = 7.8 Hz, H-1’); 2.26 (s, 6H, CH_3_CO); 2.05 (s, 3H, CH_3_CO); 1.98 (m, 2H, H-2’). ^13^C-NMR: 171.1 (CH_3_CO); 168.4 (CH_3_CO); 168.3 (CH_3_CO); 141.9 (C-2); 140.2 (C-1); 140.1 (C-4); 126.5 (C-5); 123.2 (C-3); 123.1 (C-6); 63.6 (C-3’); 31.6 (C-2’); 29.9 (C-1’); 20.9 (CH_3_); 20.6 (2 × CH_3_). Compound **7**; IR (cm^−1^): 2943 (C-H); 1707 (C=O); 1429 (-CH_2_); 1371 (-CH_3_); 1221 (C-O); 1017 (C-O); 905 (C-H). M.S. (m/z, %): 236 (5.3); 194 (19.3); 153 (11.8); 152 (100); 124 (6.9); 123 (74.5); 109 (3.6); 77 (5.4). ^1^H-NMR: 7.06 (m, 2H, H-5 and H-6); 6.99 (d, 1H, *J* = 1.6 Hz, H-3); 2.57 (t, 2H, *J* = 7.7 Hz, H-1’); 2.28 (s, 6H, CH_3_CO); 1.63 (m, 2H, H-2’); 0.95 (t, 3H, *J* = 7.7 Hz, H-3’). ^13^C-NMR: 168.5 (CH_3_CO); 168.4 (CH_3_CO); 141.7 (C-2); 141.6 (C-1); 139.8 (C-4); 126.5 (C-5); 123.1 (C-6); 122.9 (C-3); 37.3 (C-2’); 24.2 (C-2’); 20.6 (2x CH_3_); 13.8 (CH_3_).

Synthesis of 4-(3-hydroxypropyl)-5-nitrobenzene-1,2-diol (**8**), 4-(2-hydroxypropyl)-5-nitrobenzene-1,2-diol (**9**), 4-[3-(acetyloxy)propyl]-5-nitro-1,2-phenylene diacetate (**10**) and 3-(4,5-dihydroxy-2-nitrophenyl)propyl acetate (**11**) from **2**: The compound **2 **(4-allyl-5-nitrobenzene-1,2-diol previously synthesized [8,9]) (0.7 g, 0.4 mmol), was cooled at −5 °C, under an atmosphere of N_2 _and slowly hydroborated with a 2.0 M solution of BH_3_·DMS/THF (0.20 mL) added dropwise at −10 °C for 15 min, and later the mixture was stirred at room temperature for 1 h. The resultant organoborane was oxidized by a solution of sodium perborate (0.10 g, 0.70 mmol) and water (100 mL) and then the mixture was stirred at room temperature for 2 h. Then, it was extracted with portions of ethyl ether (4 × 50 mL) and the layers were separated. The organic layer was dried over MgSO_4_, filtered, evaporated and re-dissolved in 5 mL of CH_2_Cl_2_. It was absorbed on silica, chromatographed by CC eluting with mixtures of petroleum ether/EtOAc of increasing polarity (16.0:4.0→14.0:6.0). Isolated two solids compounds. Solids were recrystallized in a mixture methanol/ether, purifying the product **8** (37.5 mg, 49.3%) and isomer **9** (4.6 mg, 6.1%). Compound **8**, dark yellow, p.f.: 98.9–99.8 °C. IR (cm^−1^): 3458 (O-H); 2921 (=C-H); 1593 (C=C); 1532 (NO_2_); 1457 (CH_2_); 1385 (CH_3_); 1331 (N=O); 880 (-C-H). ^1^H- NMR: 9.24 (s, 1H, OH); 8.88 (s, 1H, OH); 7.53 (s, 1H, H-6); 6.85 (s, 1H, H-3); 5.89 (b.s, 1H, OH); 4.39 (t, 2H, J = 6,4 Hz, H-3’); 2.88 (m, 2H, H-1’), 1.79 (m, 2H, H-2’). ^13^C-NMR: 151.3 (C-2); 144.2 (C-1); 141.5 (C-5); 132.2 (C-4); 118.5 (C-3); 112.9 (C-6); 61.8 (C-3’); 34.4 (C-2’); 30.1 (C-1’). Compound **9**, dark yellow; p.f.: 104.3–105.8 °C. IR (cm^−1^): 3520 (O-H); 2923 (C-H); 1599 (C=C); 1530 (NO_2_); 1458 (-CH_2_); 1376 (-CH_3_); 1327 (N=O); 1043 (-C-O); 885(-C-H). ^1^H-NMR: 9.19 (s, 1H, OH); 8.84 (s, 1H, OH); 7.53 (s, 1H, H-6); 6.84 (s, 1H, H-3); 4.12 (m, 1H, H-2’); 3.73 (b.s., 1H, OH); 3.36 (dd, 1H, J = 13.6, 3.9 Hz, H-1’_a_); 3.16 (dd, 1H, J = 13.6, 8.4 Hz, H-1’_b_); 1.33 (d, 3H, J = 6.6 Hz, H-3’). ^13^C-NMR: 151.1 (C-2); 145.1 (C-1); 144.2 (C-5); 125.8 (C-4); 114.6 (C-3); 108.55 (C-6); 68.4 (C-2’); 39.1 (C-1’), 24.7 (C-3’). Then, this complex mixture (0.15 g) was dissolved in dry CH_2_Cl_2_ (50 mL), DMAP (1.08 mg) and Ac_2_O (0.40 mL, 4.23 mmol) were added and the mixture was stirred at room temperature for 2 h. A cooled solution of 10% KHSO_4_ (approx. 50 mL) was then added to this mixture. The watery layer was discarded and the organic layer was washed to neutrality with a saturated solution of NaHCO_3_ and water. Then it was dried over MgSO_4_, filtered, evaporated and re-dissolved in 5 mL of CH_2_Cl_2_. Subsequently, it was absorbed on silica and chromatographed by CC with petroleum ether/EtOAc mixtures of increasing polarity (18.8:1.2→15.6:4.4). Two products were isolated and identified as nitrated triacetyl derivative **10** (139.2 mg, 44.1%), and monoacetylated nitrated compound **11** (7.2 mg, 3.0%). Compound **10**, IR (cm^−1^): 2955 (C-H); 1757 (C=O); 1588 (C=C); 1531 (NO_2_); 1432 (-CH_2_); 1368 (C-NO_2_); 1045 (C-O). M.S. (m/z, %): 339 (2.8); 326 (5.7); 298 (23.3); 267 (8.2); 255 (18.3); 199 (11.3); 143 (25.3); 87 (69.6); 74 (100); 69 (14.1); 55 (22.0). ^1^H-NMR: 7.89 (s, 1H, H-6); 7.23 (s, 1H, H-3); 4.14 (t, 2H, J = 6.2 Hz, H-3’); 2.98 (t, 2H, J = 7.8 Hz, H-1’); 2.32 (s, 6H, CH_3_CO); 2.06 (s, 3H, CH_3_CO); 2.00 (m, 2H, H-2’). ^13^C-NMR: 171.1 (CH_3_CO); 167.6 (CH_3_CO); 167.3 (CH_3_CO); 145.9 (C-5); 145.6 (C-2); 140.4 (C-1); 135.8 (C-4); 126.4 (C-3); 120.9 (C-6); 63.5 (C-3’); 29.7 (C-2’); 29.2 (C-1’); 20.9 (CH_3_CO); 20.6 (CH_3_CO); 20.5 (CH_3_CO). Compound **11**; IR (cm^−1^): 3351 (O-H); 2978 (C-H); 1739 (C=O); 1633 (C=C); 1546 (NO_2_); 1425 (CH_2_); 1253 (C-O). ^1^H-NMR: 7.56 (s, 1H, H-6); 6.85 (s, 1H, H-3); 4.06 (t, 2H, J = 6.4 Hz, H-3’); 2.94 (b.s., 2H, 2×OH); 2.92 (t, 2H, J = 7.6 Hz, H-3’); 1.99 (s, 3H, CH_3_CO); 1.91 (m, 2H, H-2’). ^13^C-NMR: 171.0 (CH_3_CO); 151.5 (C-2); 144.4 (C-1); 141.4 (C-5); 131.4 (C-4); 118.7 (C-3); 113.2 (C-6); 64.1 (C-3’); 30.3 (C-2’); 29.9 (C-1’); 20.7 (CH_3_CO).

### 3.2. Cell Lines

The experimental cell cultures were obtained from American Type Culture Collection (Rockville, MD, USA). MCF-7, MDA-MB-231 cells were grown in DMEM-F12 medium containing 10% FCS, 100 U/mL penicillin, 100 µg/mL streptomycin and 1 mM glutamine. Cells are seeded into 96 well microtiter plates in 100 µL at plating density of 3 × 10^3^ cells/well. After 24 h incubation at 37 °C (under a humidified 5% carbon dioxide to allow cell attachment) the cells were treated with different concentrations of drugs (safrole and derivatives) and incubated for 72 h under the same conditions. Stock solution of compounds was prepared in ethanol and the final concentration of this solvent was kept constant at 0.1%. Control cultures received 0.1% ethanol alone.

### 3.3. *In vitro* Growth Inhibition Assay

The sulforhodamine B assay according to the method of Skehan *et al.* [22] was used with some modifications [23]. Briefly, the cells were set up 3 × 10^3^ cells per well of a 96-well, flat-bottomed 200 μL microplate. Cells were incubated at 37 °C in a humidified 5% CO_2_/95% air mixture and treated with the compounds at different concentrations for 72 hours. At the end of drug exposure, cells were fixed with 50% trichloroacetic acid at 4 °C (TCA final concentration 10%). After washing with destilled water, cells were stained with 0.1% sulforhodamine B (Sigma-Aldrich, St. Louis, MO, USA), dissolved in 1% acetic acid (50 µL/well) for 30 min, and subsequently washed with 1% acetic acid to remove unbound stain. Protein-bound stain was solubilized with 100 µL of 10 mM unbuffered Tris base. The cell density was determined using a fluorescence plate reader (wavelength 540 nm). Values shown are the mean + SD three independent experiments in triplicate.

## 4. Conclusions

In synthetic terms, the establishment of a new protocol for the deprotection of safrole with BF_3_ was achieved with good performance and successful hydroboration. In relation to the above method, both protected and free catechols enhanced the reaction in order to resolve the complex mixture. As a subproduct, a catechol with an *n*-propyl side chain was unexpectedly obtained. The safrole derivatives **6 ** and **10 ** proved to be more cytotoxic than safrole and derivative **2** against the selected cancer cells lines. These results indicate that **6 ** and **10** exert their cytotoxic and selective effects on the breast cancer cells lines MCF-7, MDA-MB-231 over a wide concentration range without effects in non tumoral cell line DFH. We think that the cytotoxic effect of catechol derivatives could be related to changes in intracellular free Ca^2+^ levels, as reported in safrole by Jan [24]. The authors are currently investigating the mechanisms of action for these catechols.
